# The altered gut microbiota in adults with cystic fibrosis

**DOI:** 10.1186/s12866-017-0968-8

**Published:** 2017-03-09

**Authors:** D.G. Burke, F. Fouhy, M. J. Harrison, M. C. Rea, P. D. Cotter, O. O’Sullivan, C. Stanton, C. Hill, F. Shanahan, B. J. Plant, R. P. Ross

**Affiliations:** 1 Teagasc Food Research Centre, Moorepark, Fermoy, Co, Cork, Ireland; 2 APC Microbiome Institute, University College Cork, Cork, Ireland; 3 HRB Clinical Research Facility, University College Cork, Cork, Ireland; 4 Cork Cystic Fibrosis Centre, University College Cork, Cork University Hospital, Wilton, Cork, Ireland; 5 School of Microbiology, University College Cork, Cork, Ireland; 6 Department of Medicine, University College Cork, National University of Ireland, Cork, Ireland; 7 College of Science, Engineering and Food Science (SEFS), University College Cork, Cork, Ireland

**Keywords:** Cystic fibrosis, Gut microbiota, 454-pyrosequencing, Microbial diversity, Antibiotic therapy

## Abstract

**Background:**

Cystic Fibrosis (CF) is an autosomal recessive disease that affects the function of a number of organs, principally the lungs, but also the gastrointestinal tract. The manifestations of cystic fibrosis transmembrane conductance regulator (CFTR) dysfunction in the gastrointestinal tract, as well as frequent antibiotic exposure, undoubtedly disrupts the gut microbiota. To analyse the effects of CF and its management on the microbiome, we compared the gut microbiota of 43 individuals with CF during a period of stability, to that of 69 non-CF controls using 454-pyrosequencing of the 16S rRNA gene. The impact of clinical parameters, including antibiotic therapy, on the results was also assessed.

**Results:**

The CF-associated microbiome had reduced microbial diversity, an increase in *Firmicutes* and a reduction in *Bacteroidetes* compared to the non-CF controls. While the greatest number of differences in taxonomic abundances of the intestinal microbiota was observed between individuals with CF and the healthy controls, gut microbiota differences were also reported between people with CF when grouped by clinical parameters including % predicted FEV_1_ (measure of lung dysfunction) and the number of intravenous (IV) antibiotic courses in the previous 12 months. Notably, CF individuals presenting with severe lung dysfunction (% predicted FEV_1_ ≤ 40%) had significantly (*p* < 0.05) reduced gut microbiota diversity relative to those presenting with mild or moderate dysfunction. A significant negative correlation (−0.383, Simpson’s Diversity Index) was also observed between the number of IV antibiotic courses and gut microbiota diversity.

**Conclusions:**

This is one of the largest single-centre studies on gut microbiota in stable adults with CF and demonstrates the significantly altered gut microbiota, including reduced microbial diversity seen in CF patients compared to healthy controls. The data show the impact that CF and it's management have on gut microbiota, presenting the opportunity to develop CF specific probiotics to minimise microbiota alterations.

**Electronic supplementary material:**

The online version of this article (doi:10.1186/s12866-017-0968-8) contains supplementary material, which is available to authorized users.

## Background

The healthy human gut is populated with in excess of 10^11^ microbes per millilitre of luminal content [1]. This population is generally stable over time in healthy adults, however, alteration of the normal gut microbiota has been seen in a number of diseases/conditions, including diabetes, obesity, irritable bowel syndrome, and inflammatory bowel disease [2–6]. More recently, it has been shown that those with cystic fibrosis (CF) also exhibit an altered gut microbiota, compared to healthy controls [7].

CF is the most common life shortening autosomal recessive disease. It is caused by a mutation in the cystic fibrosis transmembrane conductance regulator (CFTR) gene, which results in expression of dysfunctional Cl^−^ ion transport proteins in epithelial cells. This causes inadequate hydration in the lumen of tubular organs, resulting in viscous mucus accumulating along a variety of epithelial surfaces, including the lungs and gastrointestinal tract [8]. CF results in a range of gastrointestinal complications including pancreatic insufficiency (PI), slowed intestinal transit, malabsorption and obstruction [9–14]. Progressive pulmonary disease due to chronic and recurrent infection is the number one cause of death in people with CF [15]. Consequently, people with CF are frequently treated with broad spectrum antibiotics [16, 17]. Previous studies in non-CF individuals have shown that antibiotic therapy reduces the diversity of the intestinal microbiota, as well as altering the relative abundances of susceptible bacterial species [18, 19]. While the intestinal microbiota has been shown to return to a normal pre-treatment state within weeks of cessation of antibiotic therapy, it has been noted that some taxonomic changes can persist for long periods after antibiotic treatment [19–22]. Frequent courses of antibiotics are also likely to increase the risk of developing antibiotic resistant strains, as well as reducing the hosts’ colonization resistance allowing bacteria to establish, increasing the risk of proliferation of opportunistic pathogens, including *Clostridium difficile* [23, 24]. Research on the impact of CF on gut microbiota has increased in recent years. Previous investigations revealed that children with CF had lower species diversity and lower temporal stability in their gut microbiota relative to non-CF sibling controls [25]. Frequent antibiotic therapy to treat pulmonary infections, in addition to the inherent effect of CFTR dysfunction on the gastrointestinal tract, have been proposed as possible causes of this altered gut microbiota of people with CF [25]. This theory is supported by studies in murine models of CF that have demonstrated decreased richness, evenness, and diversity of the small intestinal microbiota relative to non-CF mice [26]. A study examining the development of the gut and lung microbiome in children with CF, revealed both microbial communities develop simultaneously and share a number of colonising species [27]. It was also revealed that the appearance of some species in the gut can presage their appearance in the lungs, suggesting the gut microbiota may help shape the development of the lung microbiota. This coupled with the success of probiotic trials at reducing gastrointestinal inflammation and exacerbation frequency in people with CF [10, 28, 29], highlights the importance of understanding the CF gut microbiota and the effect of disease manifestation and its treatment on this ecosystem.

To date, studies investigating the CF gut microbiota have varied in approach adopting both culture-dependent and culture-independent approaches in either children with CF [25, 30] or CF animal models [26, 31]. In this study, the effect of CF combined with its treatment on the gut microbiota of 43 adults with CF was investigated using high-throughput 454-pyrosequencing. The results of this study demonstrated that the gut microbiota of adults with CF is significantly altered relative to that of the non-CF control group. Gut microbiota diversity also correlated with several clinical parameters, most notably antibiotic exposure. This research on the gut microbiota of CF adults is highly pertinent given the change in the CF cohort age profile. As CF patients live longer, there is a need to understand the impact that long-term exposure to CF therapies, including antibiotics, have on an adult gut microbiota, with the future aim of minimising any microbiota disturbances via probiotic interventions, to achieve a gut microbiota comparable with a healthy cohort.

## Methods

### Study participants

A total of 43 individuals with CF (25 males;18 females, Mean age of all CF individuals, 29 ± 8.3 years; median age, 27 years) were recruited during a period of stability (no changes to their pulmonary status as determined by their clinical team) from the Cork Adult Cystic Fibrosis Centre, Cork University Hospital. No participants reported acute or active gastrointestinal symptoms at the time of sampling. One faecal sample was collected per patient, upon visit to the CF clinic. Individuals who were undergoing a pulmonary exacerbation (as determined by their clinical team) at the time of sampling or those who had received a lung transplant were excluded from the study. A total of 69 non-CF volunteers (*n* = 49 males; 20 females, Mean age of all controls, 32 years ± 8.1 years; median age, 30 years) were recruited from the greater Cork area as a control group for comparison of their gut microbiota, to that of individuals with CF. Non-CF volunteers were eligible for inclusion provided they were aged 20–80 years, reported no gastrointestinal illness at the time of sampling, and had not received antibiotics within the previous 6 months. This study was approved by the Clinical Research Ethics Committee of the Cork Teaching Hospitals (reference number ECM 4 (kk) 01/05/12). Written informed consent was obtained from all subjects in accordance with local research ethics committee guidelines.

### Pyrosequencing and bioinformatics

Metagenomic DNA was extracted from faecal samples using the QIAamp DNA Stool Mini Kit, as per the manufacturer’s instructions (Qiagen, Crawley, West Sussex, UK) with the addition of an initial bead-beating step (30s beating followed by 30s on ice repeated for a total of 3 min using 3 mm, 1 mm and 0.1 mm zirconia/silica beads; Stratech Scientific). Samples were prepared for compositional sequencing by amplification of the V4 region (239 bp product) of the 16S rRNA gene using universal 16S primers one forward, i.e. F1 (5′ AYTGGGYDTAAAGNG), and a combination of 4 reverse primers, R1 (5′ TACCRGGGTHTCTAATCC), R2 (5′ TACCAGAGTATCTAATTC), R3 (5′ CTACDSRGGTMTCTAATC) and R4 (5′ TACNVGGGTATCTAATC) as described by Ribosomal Database Projects (RDP) Pyrosequencing pipeline (http://pyro.cme.msu.edu/pyro/help.jsp) [32, 33]. Samples were individually barcoded to enable pooling of samples for sequencing and subsequent bioinformatic identification. Negative controls (where UV treated PCR-grade water replaced DNA in the PCR reactions) were included for all PCRs to ensure reactions were free from contamination. The resulting amplicons were sequenced using a Roche 454 GS FLX, at the Teagasc sequencing facility. Raw sequences were quality trimmed using QIIME [34]. Reads with a minimum quality score of 25 and sequence lengths shorter than 150 bp for 16S rRNA amplicon reads were removed. Trimmed FASTA sequences were BLASTed against the SILVA 16S rRNA database (version 106). The resulting BLAST outputs were parsed using MEGAN [35], which assigns reads to NCBI taxonomies using the Lowest Common Ancestor algorithm. A bit cut-off score of 86 was used to filter the results prior to tree construction. Phylum, family, and genus counts for each individual were extracted from MEGAN. Operational taxonomic unit (OTU) assignment, chimera checking, clustering and α (Chao1, Shannon diversity index, Simpson diversity index and number of observed species tests) and β diversity analysis (weighted and unweighted Unifrac) were implemented with QIIME. Principal coordinate analysis (PCoA) plots were visualised using EMPeror v0.9.3-dev.

### Statistical analysis

Statistical analysis was performed on the sequencing data using the non-parametric Kruskal-Wallis test in Minitab Release 15.1.1.0 (Minitab Inc., 2007). Statistical significances were accepted at *p* < 0.05, adjusted for ties. Correlation analysis was completed using the Spearman Rank correlation 2 tailed test using GraphPad Prism 5, where significant correlations were accepted as *p* < 0.05. Statistical analysis was performed to compare the results between the CF patients and the controls. Additionally we examined the impact of a number of clinical parameters such as *Clostridium difficile* carriage, lung function and antibiotic usage, on the CF gut microbiota.

## Results

### Gut microbiota analysis

#### Gut microbiota diversity analysis of individuals with CF compared to non-CF controls

The gut microbiota of individuals with CF and non-CF controls was investigated using high-throughput 16S rRNA gene amplicon sequencing of faecal samples. A total of 2,099,804 reads were sequenced, corresponding to an average 23,331 reads/sample. Alpha and beta diversity analysis was completed to determine the gut microbiota diversity of the CF samples, compared to the non-CF controls.

**Fig. 1:**
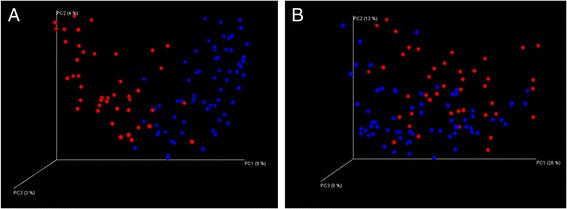
Visualisation of the PCoA analysis based on unweighted (**a**) and weighted (**b**) Unifrac distance matrices. Samples separate into 2 clusters, CF samples (*red*) and control samples (*blue*)

The gut microbiota of those with CF was found to be significantly (*p* < 0.05) less diverse compared to the non-CF controls (Additional file 1: Figure S1). Principal coordinate analysis plots generated using unweighted (A) and weighted (B) Unifrac distance matrices showed that those with CF clustered separately to non-CF controls (Fig. 1). However, individuals with CF did not cluster based on carriage of *Clostridium difficile*, class of CFTR mutation, % predicted FEV_1_, pancreatic insufficiency, inpatient days or treatments received i.e. proton pump inhibitors, courses of IV antibiotics and macrolide antibiotic therapy (data not shown). This suggests CF and its associated therapy has a greater combined effect on the diversity of the gut microbiota than any individual treatment.

#### Gut microbiota composition of individuals with CF compared to non-CF controls

**Fig. 2:**
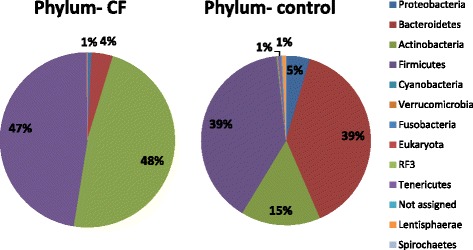
Percentage relative abundance of phyla in those with CF compared to non-CF controls

Assigned sequence reads were used to assess differences in taxonomic abundances between those with CF and healthy controls at phylum, family, and genus levels. There was a significant (*p* < 0.05) decrease in the relative abundance of *Bacteroidetes, Proteobacteria*, *Cyanobacteria*, *Verrucomicrobia*, *RF3*, *Tenericutes*, and *Lentisphaerae* in individuals with CF at the phylum level, relative to the non-CF controls (Fig. 2). Notably, there were significant (*p* < 0.05) increases in *Firmicutes* and *Actinobacteria* in people with CF relative to the controls (47% vs. 39% respectively). At phylum level, the CF gut microbiota was dominated by *Actinobacteria* (48%) and *Firmicutes* (47%) compared to the non-CF controls, in whom *Bacteroidetes* accounted for 39% of phyla reads, compared to just 4% in the CF study group.

**Fig. 3:**
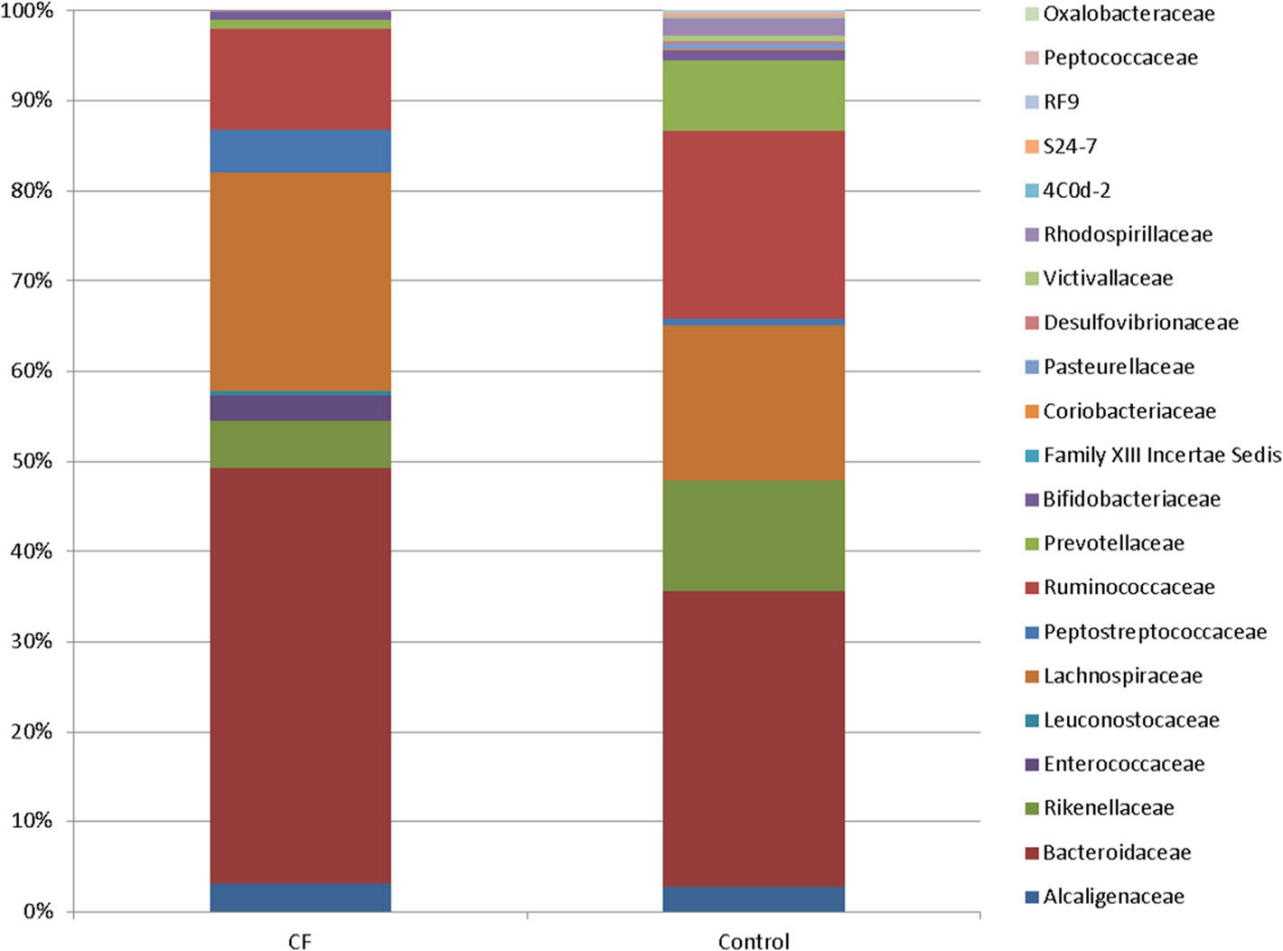
Percentage relative abundance of the 21 families that were significantly different in the CF study cohort compared to the non-CF controls

At the family level, a total of 21 families were present at significantly altered proportions (*p* < 0.05) in those with CF compared to non-CF controls; 14 families decreased including, *Alcaligenaceae*, *Prevotellaceae*, *Bifidobacteriaceae* and *Peptococcaceae* (Fig. 3). At the genus level, significant differences in 42 genera were detected in those with CF compared to non-CF controls (data not shown). This included increased *Enterococcus*, *Bacteroides*, *Leuconostoc* and decreased *Roseburia*, *Prevotella*, *Odoribacter*, *Faecalibacterium* and *Bifidobacterium*.

### CF gut microbiota alterations based on clinical parameters

#### The effect of duration of intravenous (IV) antibiotic treatment on gut microbiota

**Fig. 4:**
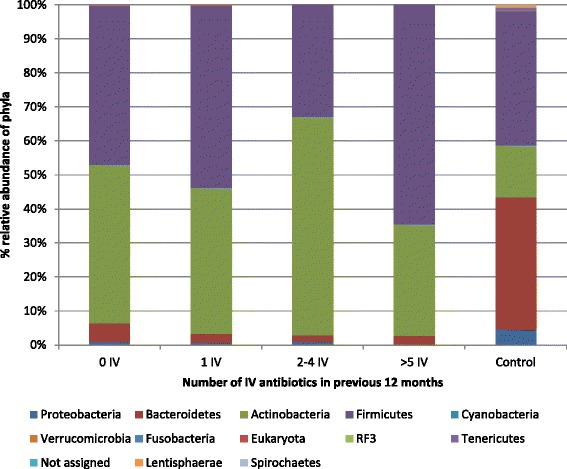
Percentage relative abundance of phyla in the non-CF controls compared to the individuals with CF, stratified based on number of IV courses in the previous 12 months

To examine the effect of IV antibiotic treatment duration on the gut microbiota, individuals with CF were divided into no (*n* = 22), low (≤1; *n* = 9) intermediate (2–4; *n* = 8) and high IV antibiotic treatment groups (≥5; *n* = 4) based on the number of courses of IV antibiotics in the previous year. The gut microbiota diversity of those with CF within the low IV antibiotic group was significantly higher compared to those in the intermediate or high IV antibiotic groups (*p* < 0.05). At phylum level, individuals with CF in the highest IV antibiotic group had the lowest *Bacteroidetes* and highest *Firmicutes* proportions of all CF individuals (Fig. 4). Two families were significantly altered in those with CF who were in the high IV antibiotic treatment group, *Veillonellaceae* being highest in these individuals and *Helicobacteriaceae* only being detected in the high IV antibiotic treated CF individuals. When the genera were compared between the controls and the CF cohort receiving 0, 1, 2–4 or ≥5 IV courses, it was evident that those receiving the greatest number of IV courses had the most altered gut microbiota compared to the non CF-controls (Additional file 2: Figure S2).

**Fig. 5:**
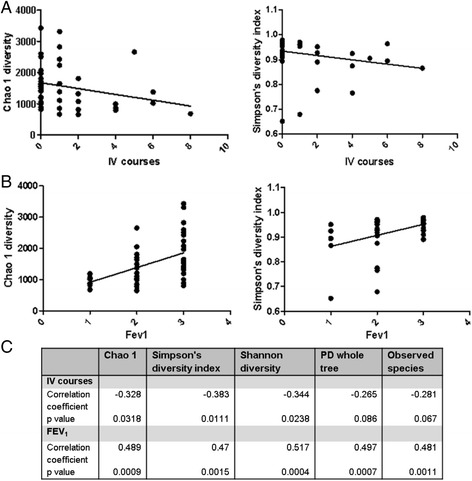
Correlation analysis of gut microbiota diversity and IV antibiotic courses (**a** Chao 1 and Simpson’s diversity index) and % predicted FEV_1_ (**b** Chao 1 and Simpson’s diversity index; 1 = FEV_1_ ≤ 40%, 2 = FEV_1_ 41–69%, 3 = FEV_1_ ≥ 70%). Panel **c** provides the correlation coefficients and *p* values for all diversity analysis

To further investigate this, we performed correlation analysis, the results of which indicated that a significant negative correlation exists between the number of IV antibiotic courses and gut microbiota diversity (Fig. 5 a/c).

#### Alteration of gut microbiota with decreasing % predicted FEV_1_

**Fig. 6:**
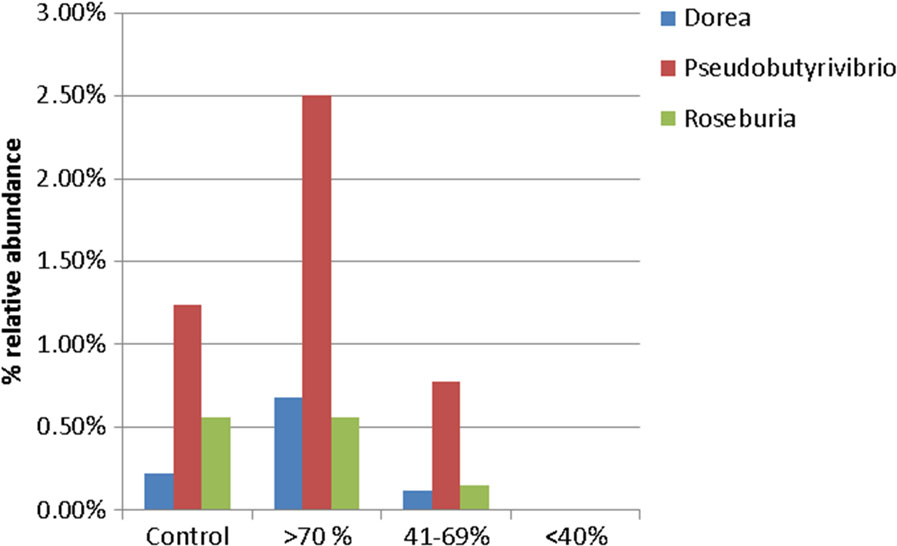
Percentage relative abundance of significantly different genera in the controls versus the individuals with CF stratified by % predicted FEV_1._ Mild lung disease was classed as % predicted FEV_1_ ≥ 70%; moderate as 41–69% and severe ≤40%

Forced expiratory volume in 1 s (FEV_1_) is a measure of lung function used to measure the progression of pulmonary disease in people with CF. To assess whether the gut microbiota changes with progression of lung disease in those with CF, we compared the gut microbiota of the CF study cohort who were stratified into having mild (≥70%; *n* = 21), moderate (41–69%; *n* = 16) or severe (≤40%; *n* = 6) lung disease [36, 37], based on their % predicted FEV_1_. The gut microbiota diversity of individuals was also correlated with % predicted FEV_1_ stratifications of mild, moderate or severe lung disease, with the results indicating that a positive correlation exists between gut microbiota diversity and % predicted FEV_1_ (Fig. 5 b/c). Individuals with CF who had severe lung dysfunction (% predicted FEV_1_ ≤ 40%) had significantly (*p* < 0.05) reduced α diversity relative to those with mild or moderate lung dysfunction; those with mild lung disease (% predicted FEV_1_ ≥ 70%) had the highest α diversity of all CF individuals studied (Fig. 5b). FEV_1_ levels appear to have the greatest impact on overall gut α diversity, rather than on any one bacterial population. When individuals with CF were stratified based on their % predicted FEV_1_, no significant differences occurred at phylum or family levels. Individuals with CF who had the highest % predicted FEV_1_ had significantly increased *Dorea*, *Pseudobutyrivibrio* and *Roseburia* at the genus level compared to those with moderate or severe lung disease (Fig. 6).

#### The effect of macrolide antibiotics on the gut microbiota of people with cystic fibrosis

The effect of continuous prophylactic macrolide antibiotic therapy on gut microbiota was investigated to determine the impact that chronic maintenance antibiotic therapy had on both the CF gut microbiota and overall diversity. Macrolide antibiotics had been used by 37 of the 43 individuals with CF in this study in the 6 months prior to sample collection. The composition of their gut microbiota was compared to those who had not received these antibiotics in the previous 6 months. Individuals receiving macrolide therapy showed no significant differences in their gut microbiota diversity (based on α diversity analysis) relative to those who had not received macrolide antibiotics. At the phylum level, those receiving macrolide antibiotics showed significant (*p* < 0.05) decreases in *Proteobacteria*, *Verrucomicrobia* and *Eukaryota*. At the family level *Akkermansiaceae, Pasteurellaceae,* and *Ruminococcaceae* were all significantly reduced in those who received macrolides in the 6 months prior to sample collection. These differences were also evident at the genus level, with significant reductions in the proportions of *Bifidobacterium* and *Akkermansia* evident in those who received macrolide antibiotics.

#### The effect of proton pump inhibitors on the gut microbiota of people with cystic fibrosis

Proton pump inhibitors (PPI) are commonly prescribed to individuals with CF to treat reflux symptoms. The effect of PPIs on the gut microbiota of people with CF was examined by comparing the gut microbiota of those on PPIs (*n* = 20) to those not on PPI therapy (*n* = 23). There was no significant (*p* > 0.05) difference in the gut microbiota diversity or bacterial populations at phylum or family levels in people with CF receiving PPI therapy, relative to those not receiving PPIs. Significant decreases in the proportions of *Dialister* and *Subdoligranulum* occurred at the genus level in CF individuals receiving PPI therapy, while *Butyricoccus* was only detected in those receiving PPIs. Thus, the overall effects of PPIs on gut microbiota were minimal.

#### Changes in the gut microbiota of pancreatic insufficient (PI) CF individuals

PI is a common condition in people with CF. This study examined the effect of PI on the gut microbiota of people with CF, by comparing the gut microbiota of those with (*n* = 35) and without PI (*n* = 8), to determine if alterations to digestion and the need for pancreatic enzyme replacement therapies in cases of pancreatic insufficiency, would impact on gut microbiota. Pancreatic sufficiency status had been previously recorded for each participant as part of routine CF care and these clinical data were made available for data analysis. No significant differences in diversity or species richness were observed between pancreatic insufficient and pancreatic sufficient CF individuals. At the phylum level, *Eukaryota* were only detected in one individual who was pancreatic sufficient, but was not detected in those who were insufficient. *Ruminococcaceae* and *Coriobacteriaceae* were significantly decreased in pancreatic insufficient CF individuals compared to those who were pancreatic sufficient. At the genus level, pancreatic sufficient CF individuals had significantly (*p* < 0.05) increased proportions of *Ruminococcus* and *Anaerotruncus,* relative to pancreatic insufficient CF individuals.

#### Impact of cystic fibrosis conductance regulator class mutation on the composition of the gut microbiota

CF is caused by a mutation in the CFTR gene which codes for a chloride ion transport protein expressed at epithelial cell surfaces. Mutations in the CFTR gene are classified into 6 classes according to the mechanism by which they disrupt the action of the CFTR protein. Class 1–3 mutations are considered the most severe as they tend to result in a complete loss of function. To assess the impact of CFTR mutations on gut microbiome composition, the gut microbiota of those with CFTR mutations from classes 1–3 (*n* = 34) was compared to those with less severe mutations (*n* = 9). No significant differences in species richness or microbial diversity were observed between the CF cohort with class 1–3 mutations and CF individuals with mutations from other classes. People with CF with class 1–3 mutations had no significant differences in the composition of their gut microbiota at phylum level. At the family level, *Enterococcaceae* was significantly (*p* < 0.05) increased, while *Ruminococcaceae* was significantly (*p* < 0.05) decreased, relative to those with less severe mutations. At the genus level, *Subdoligranulum*, *Paraprevotella*, *Anaerotruncus*, and *Barnesiella* were all significantly (*p* < 0.05) lower in those with severe mutations (classes 1–3).

#### Alterations in the gut microbiota of CF individuals carrying C. difficile

To assess whether alterations in the gut microbiota may predispose people with CF to colonisation with *C. difficile,* we compared the gut microbiota of people with CF who were asymptomatic carriers of *C. difficile* (*n* = 20), to those negative for *C. difficile* carriage (*n* = 13). *C. difficile* carriage was determined as outlined in our previously published work [38]. A non-significant (*p >* 0.05) reduction in microbial diversity was observed in CF individuals carrying *C. difficile.* Those positive for *C. difficile* carriage had a significant (*p* < 0.05) decrease in the proportions of *Proteobacteria* at the phylum level relative to *C. difficile* free CF individuals. At the family level, significant increases in *Erysipelotrichaceae Incertae Sedis* were observed in CF individuals carrying *C. difficile*. At the genus level, no significant differences occurred between those carrying *C. difficile* and those free of *C. difficile*.

## Discussion

This study used high-throughput pyrosequencing of the faecal microbiota of stable CF patients to investigate the impact of CF on the gut microbiota compared to non-CF controls. In addition, using clinical data, it was investigated whether certain parameters including class of CF mutation, antibiotic exposure and FEV_1_ values were associated with alterations to the gut microbiota in individuals with CF.

The intestinal microbiota of subjects with CF was shown to be altered compared to that of healthy controls, as evidenced by their separate clustering by PCoA. The diversity of the gut microbiota of individuals with CF was also significantly reduced compared to that of healthy controls. This is a common finding of studies on CF gut microbiota compared to controls [39, 40]. Indeed, it appears from studies on children with CF that this decrease in diversity occurs early in life for CF patients, with diversity decreasing with age, with CF teenagers having diversity levels similar to those seen in healthy control infants (1 year) [39]. As CF patients live longer, there will be an increased need to appreciate how this diversity decreases with age and how best to minimise this decline, to maximise patient gut microbiota diversity.

The CF samples did not cluster distinctly based on mutations, antibiotic exposure, % FEV_1_ or other clinical parameters. Thus, while CF evidently affects overall gut microbiota diversity, the main causative factor for this is unclear and most likely it is due to multiple factors, including antibiotic exposure, genotype etc. Additionally, alterations in the taxonomic abundance were observed between the two groups at the phylum, family and genus levels. For example, it was notable that there was a decrease in *Faecalibacterium*, *Roseburia* and *Bifidobacterium* in those with CF compared to the non-CF controls, as these bacteria are generally regarded as markers for a healthy gut microbiota [41–45]. Such findings support previous CF gut microbiota studies [46] including recent work by Schippa *et al*. who also noted decreased *Faecalibacterium* and *Bifidobacterium* in CF individuals [47]. Indeed, several studies have shown a decrease in *Bifidobacterium* in CF patients compared to controls. A study using a Phylochip approach found the relative abundance of *Bifidobacterium* was decreased compared to controls [40]. Additionally, that study also found that the majority of changes to the gut microbiota in CF occurred within the *Firmicutes* and *Actinobacteria* phyla, which is consistent with our findings. Decreases in the relative abundance of potentially beneficial gut microbiota populations including *Bifidobacterium*, presents the opportunity to develop probiotics for use in a CF population. Some initial studies using probiotics in people with CF have shown promising results [10, 28, 29, 48]. These studies have shown probiotic use in CF results in decreased pulmonary exacerbations, reduced hospital admissions and beneficial alterations to the gut microbiota. A recent review highlighted the beneficial effects of probiotics in CF, but also emphasises the requirement for further work on mechanisms of action and the specific strains and dosage required to be effective [49]. Despite our study being unable to identify which factor or combination of factors cause the altered gut microbiota in CF, understanding the alterations that occur in the CF gut microbiota compared to healthy controls presents the opportunity to investigate targeted therapeutic approaches.

A shift towards an increased *Firmicutes* to *Bacteriodetes* ratio was observed in CF subjects relative to the non-CF control group. Under nutrition in CF patients is associated with poor clinical outcomes, therefore those with CF are recommended to consume a diet high in fat and protein [50]. Diets high in fat have been shown to increase the *Firmicutes* to *Bacteriodetes* ratio in mice, independent of obesity [51]. However, while diet may play a role in the altered gut microbiota, it is unlikely that diet alone accounted for the overall change in microbiota. Due to a lack of dietary data in this study, we could not interrogate this further, but it is a topic we will investigate in future studies.

Another major factor that alters the gut microbiota is chronic antibiotic exposure. CF individuals are frequently exposed to macrolide therapy and IV antibiotics to treat pulmonary exacerbations. We have demonstrated a negative correlation between IV courses and gut microbiota diversity. Moreover, those receiving the greatest exposure to IV antibiotics had the highest levels of *Firmicutes* of all CF individuals studied. Interestingly, studies investigating the consequences of short-term antibiotic exposure on gut microbiota (completed in faecal fermentation models, mice and humans) have revealed a dominance of *Proteobacteria* [32, 33, 52]. Indeed, several studies of CF children have found an increase in *Proteobacteria* compared to levels seen in controls. Hoffman *et al.* found (using next-generation sequencing) a correlation between the marked increased abundance of *E. coli* and altered gut functionality in CF children compared to controls [53, 54]. Previous studies using the Illumina MiSeq sequencing platform to analyse the progression of the CF microbiota in children with CF found that the development of the CF gut microbiota is altered compared to that of healthy control children [39]. The study also noted decreased *Faecalibacterium* as was seen in our study, but found that *Escherichia/Shigella* levels increased with increasing age. Differences in levels of *Proteobacteria* seen in several studies compared to ours likely reflect the age of the subjects studied and the use of different research methods. Given the chronic exposure of the CF gut microbiota to antibiotics, findings of increased *Firmicutes* rather than *Proteobacteria* dominance in CF adults, perhaps demonstrates a state of microbiota equilibrium in those with CF, which is not only altered compared to healthy controls, but also compared to those on short-term antibiotic therapy. It has been demonstrated that repeated antibiotic therapy causes the microbiota to return to an intermediate microbiota, resembling the microbiota prior to exposure, while also retaining some features associated with antibiotic treatment [55, 56]. Studies examining how the CF gut microbiota changes from periods of stability compared to during pulmonary exacerbations (when antibiotic therapy would be increased) are required. Additionally, studies with longer follow up periods post-exacerbation would provide insight into how the CF gut microbiota recovers following intensive antibiotic exposure. We also investigated the effects of macrolide antibiotics on the gut microbiota of CF individuals and found that macrolides reduced *Bifidobacterium* and *Akkermansia*. These bacteria have been positively associated with gut health [45, 57]. It is worth noting that the majority of individuals with CF were receiving macrolide therapy in the 6 months prior to the study and so this topic should be further studied in a larger CF cohort to confirm such findings. However, the results suggest that there is an opportunity to use concomitant probiotics during antibiotic therapy to stimulate the growth of beneficial bacteria in the gut.

This study did not utilise any culture-based approaches to confirm the sequencing-based results. Previously, studies have employed culture-based approaches to investigate the airway microbiota in patients with CF [58]. However, while culture-based approaches provided insight into the lung microbiota in CF, the application of next-generation sequencing technologies advanced our appreciation of the complexity of this environment, including the prevalence of strict anaerobic bacteria, such as *Veillonella*, not previously associated with the lungs. Future studies would benefit from combining culturing and sequencing-based approaches to provide insight into the viability of the bacteria detected via sequencing-based approaches.

A positive correlation between % predicted FEV_1_ and gut microbiota diversity was identified. This is the first time that such a correlation has been reported. Percentage predicted FEV_1_ is used as a marker of lung function and therefore is used to track lung disease progression. As lung disease progresses, increased antibiotic exposure is almost guaranteed and so a collateral decrease in gut microbiota diversity is not surprising. However, identifying such a correlation presents a potential opportunity to use % predicted FEV_1_ as a marker, not only for lung function, but also as an indicator of gut microbiota diversity. Using such information could enable measures such as probiotic and/or prebiotic therapy to be applied to help minimise the decrease in gut diversity, thereby minimising the associated negative health effects.

Individuals with CF frequently suffer from PI, with >85% of individuals with CF having PI at birth [15]. This results in altered digestion, particularly a reduced ability to digest fats and to absorb fat soluble vitamins. Surprisingly, our findings indicated no significant effects of PI on gut microbiota diversity compared to pancreatic sufficient subjects and changes in relative abundance of bacterial taxa were minimal. Such findings may suggest that the use of pancreatic enzyme therapy in those with PI may help limit the effects of altered nutrient metabolism on gut microbiota diversity, thus explaining why pancreatic sufficient and insufficient individuals did not differ in terms of their gut microbiota diversity. However, as only 8 subjects were pancreatic sufficient, these findings would need to be confirmed in a larger study with greater statistical power. Additionally, this study noted that neither the class of CFTR mutation, nor the use of proton pump inhibitors affected the gut microbiota diversity. We did note that those with more severe CFTR gene mutations had significant changes in their gut microbiota compared to those with mild mutations. Differences in gut microbiota based on genotype have previously been shown, again demonstrating that more severe mutations lead to greater disturbances in the gut microbiota [47].

Despite *C. difficile* being considered an opportunistic gut pathogen, which may proliferate following antibiotic therapy, we did not observe a significant decrease in the gut microbiota diversity of those carrying *C. difficile* compared to pathogen-free individuals. However, we know from a previous study performed with this cohort that 50% of people with CF carry *C. difficile* in their stool and 63% (19/30) of the isolates were toxigenic [38]. Interestingly, while toxin was detected in the stool samples of those positive for *C. difficile*, none of these individuals reported any of the gastrointestinal symptoms normally associated with *C. difficile,* suggesting that the CF gut protected these individuals from developing *C. difficile* infection. Further studies are required to understand the reasons for the high rate of asymptomatic carriage of *C. difficile* in individuals with CF.

## Conclusions

This is one of the largest single-centre, stable adult CF cohort studies to date, to investigate the gut microbiota of individuals with CF using next-generation sequencing techniques. Additionally, we have interrogated their associated clinical history and identified which clinical aspects correlate to altered gut microbiota. We have identified the correlation between % predicted FEV_1_ and gut microbiota diversity, as well as specific bacterial populations which are reduced in those with CF compared to control subjects (many of which are associated with gut health including *Bifidobacterium* and *Akkermansia)*, revealing an opportunity for targeted probiotic therapy. These findings are important to help determine the potential opportunities to minimise the disruption to the CF gut microbiota that occurs due to disease and it's management. Understanding the changes in an adult CF population is also important as a large percentage of the studies to date have been completed in children. However, people with CF are living longer, therefore there is a greater need to understand the impact that chronic exposure to CF therapeutics, most especially antibiotics, has on the gut microbiota in an older CF population, as described in this study. We acknowledge that our study has certain limitations including an inability to investigate the impact of diet on gut microbiota due to a lack of dietary data. Additionally, individuals were only studied at one time point. Furthermore, we were unable to separate the impact of several factors on the gut microbiota and as such our findings likely reflect the impact of several factors in combination on the gut microbiota of CF patients. Our aim is to conduct further studies to interrogate the different effects (e.g. genotype, lung disease severity, etc.) on gut microbiota in future studies. Given the interesting findings from this study, further studies will be conducted to investigate the gut microbiota of an even larger cohort of subjects with CF at different time points. As our appreciation of the role of CF in the alteration of gut microbiota grows, the requirement for further knowledge is essential towards the development of new therapies to support treatment.

## Additional files

Additional file 1: Alpha diversity analysis indicating significantly decreased gut microbiota diversity in people in people with CF compared to that of the controlz. (TIF 52 kb)
Additional file 2: Percentage relative abundance of genera in the non-CF controls compared to the individuals with CF, statified based on number of IV courses in the previous 12 months. (TIF 207 kb)
