# Erratum to: The altered gut microbiota in adults with cystic fibrosis

**DOI:** 10.1186/s12866-017-1006-6

**Published:** 2017-04-27

**Authors:** D.G. Burke, F. Fouhy, M. J. Harrison, M. C. Rea, P. D. Cotter, O. O’Sullivan, C. Stanton, C. Hill, F. Shanahan, B. J. Plant, R. P. Ross

**Affiliations:** 1 Teagasc Food Research Centre, Cork, Ireland; 2 APC Microbiome Institute, Cork, Ireland; 3 HRB Clinical Research Facility, University College Cork, Cork, Ireland; 4 Cork Cystic Fibrosis Centre, University College Cork, Cork University Hospital, Cork, Ireland; 5 School of Microbiology, University College Cork, Cork, Ireland; 6 Department of Medicine, University College Cork, National University of Ireland, Cork, Ireland; 7 College of Science, Engineering and Food Science (SEFS), University College Cork, Cork, Ireland

## Erratum

In the original manuscript [1] there was a mislabelling of the data resulting in Actinobacteria and Bacteroidetes at phylum level being switched. This has been corrected in the amended Figs. 2 and 4 below where before you had high levels of Actinobacteria; these are now corrected to Bacteroidetes. These amended figures show that there was a significant (*p* < 0.05) decrease in the relative abundance of *Actinobacteria, Proteobacteria*, *Cyanobacteria*, *Verrucomicrobia*, *RF3*, *Tenericutes*, and *Lentisphaerae* in individuals with CF at the phylum level, relative to the non-CF controls (Fig. 2). Notably, there was a significant (*p* < 0.05) increase in *Firmicutes* in people with CF relative to the controls (47% vs. 39% respectively). The overall conclusions in the manuscript remain unchanged.

**Fig. 2:**
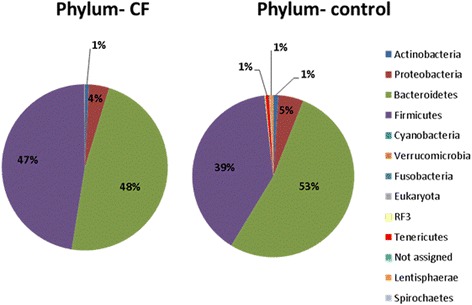
Percentage relative abundance of phyla in those with CF compared to in non-CF controls

**Fig. 4:**
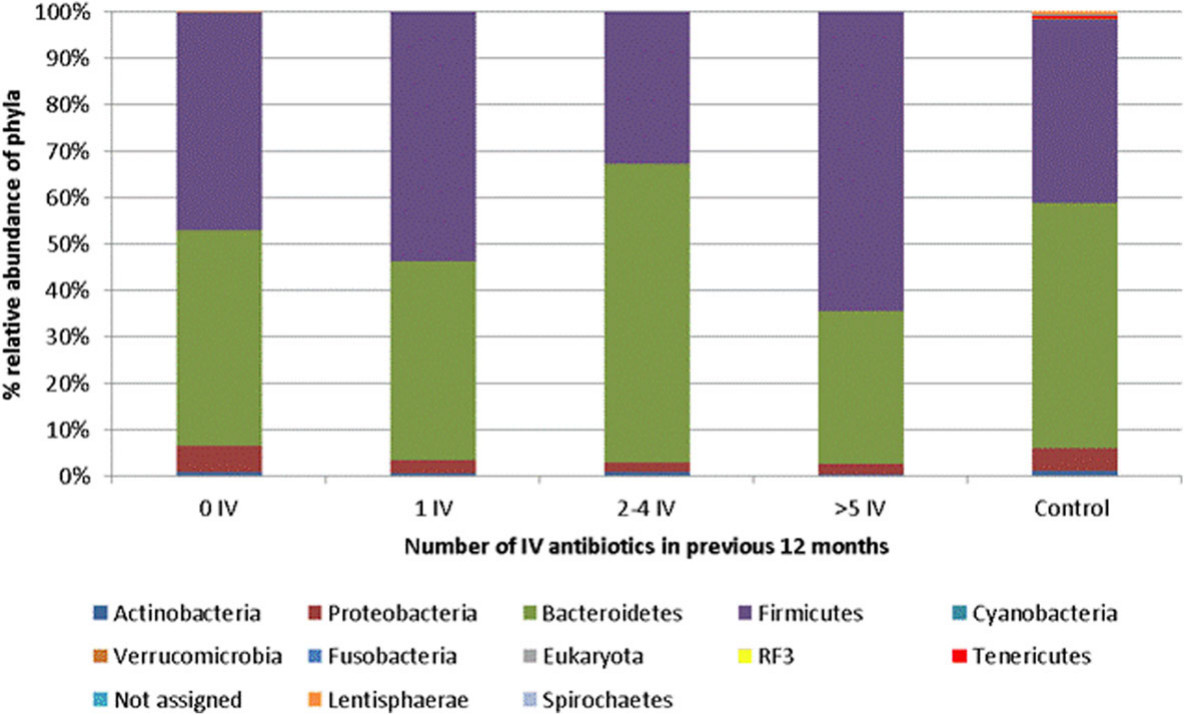
Percentage relative abundance of phyla in the non-CF controls compared to the individuals with CF, stratified based on number of IV courses in the previous 12 months
