# Supplementary figures and images for: The altered gut microbiota in adults with cystic fibrosis
Source: BMC Microbiol. 2017 Mar 9;17:58. doi: 10.1186/s12866-017-0968-8 (PMC5345154; doi:10.1186/s12866-017-0968-8)

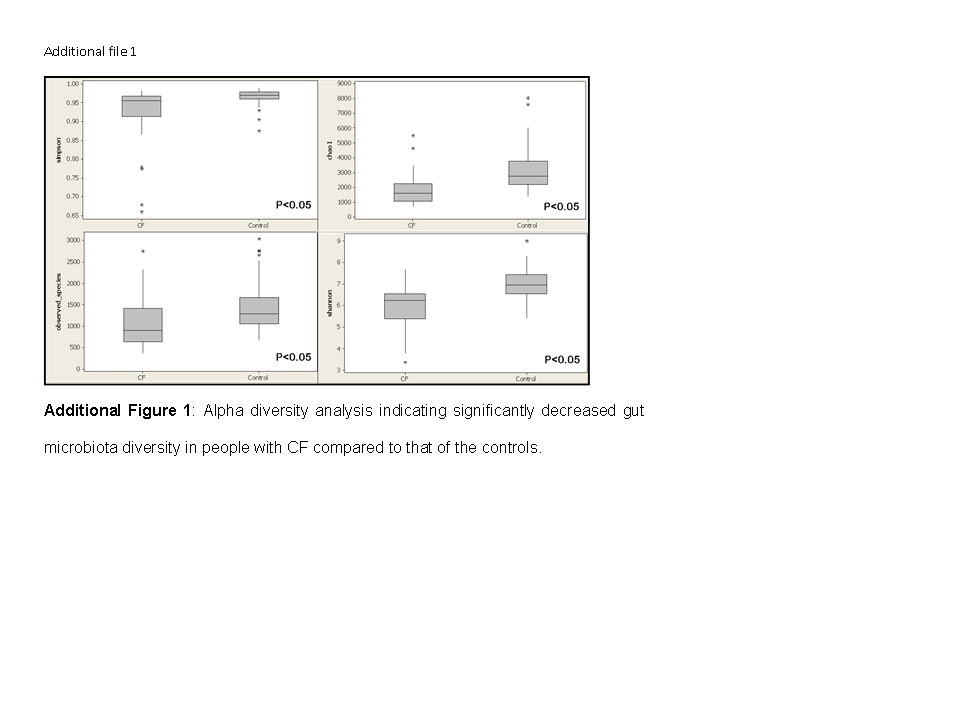

Supplement: Additional file 1: — Alpha diversity analysis indicating significantly decreased gut microbiota diversity in people in people with CF compared to that of the controlz. (TIF 52 kb) [file 12866_2017_968_MOESM1_ESM.tif]

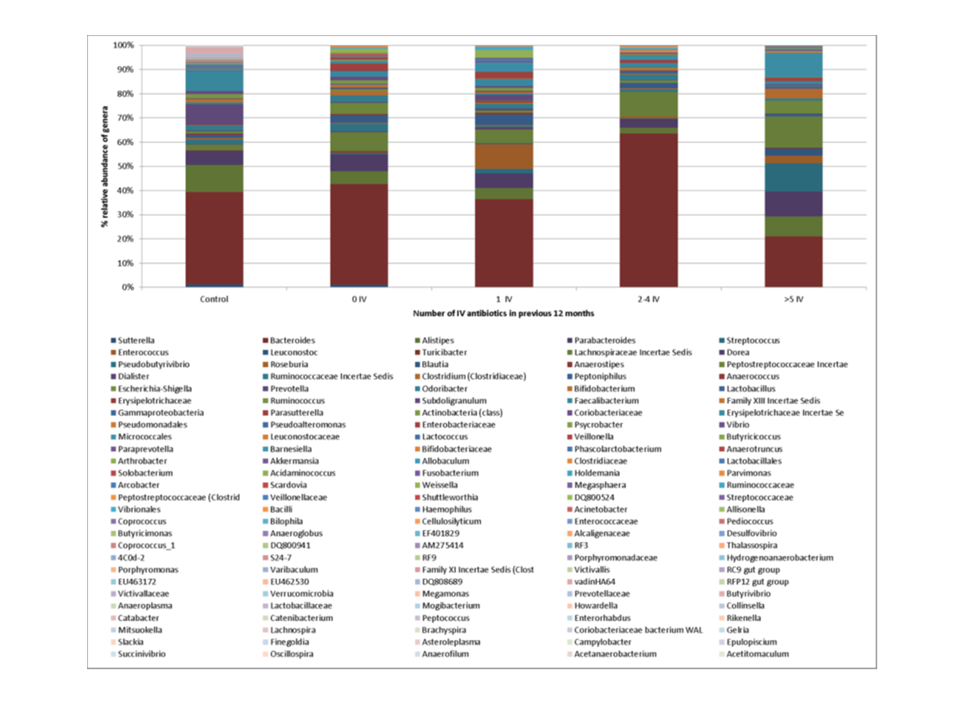

Supplement: Additional file 2: — Percentage relative abundance of genera in the non-CF controls compared to the individuals with CF, statified based on number of IV courses in the previous 12 months. (TIF 207 kb) [file 12866_2017_968_MOESM2_ESM.tif]
